# The Importance of Biophysical and Biochemical Stimuli in Dynamic Skeletal Muscle Models

**DOI:** 10.3389/fphys.2018.01130

**Published:** 2018-08-22

**Authors:** Babette Maleiner, Janine Tomasch, Philipp Heher, Oliver Spadiut, Dominik Rünzler, Christiane Fuchs

**Affiliations:** ^1^Department of Biochemical Engineering, University of Applied Sciences Technikum Wien, Vienna, Austria; ^2^The Austrian Cluster for Tissue Regeneration, Vienna, Austria; ^3^Ludwig Boltzmann Institute for Experimental and Clinical Traumatology/AUVA Research Center, Vienna, Austria; ^4^Trauma Care Consult GmbH, Vienna, Austria; ^5^Institute of Chemical Engineering, Vienna University of Technology, Vienna, Austria

**Keywords:** skeletal muscle tissue engineering, stimulation strategies, bioreactors, myokines, skeletal muscle disease models, biomaterials, myogenesis

## Abstract

Classical approaches to engineer skeletal muscle tissue based on current regenerative and surgical procedures still do not meet the desired outcome for patient applications. Besides the evident need to create functional skeletal muscle tissue for the repair of volumetric muscle defects, there is also growing demand for platforms to study muscle-related diseases, such as muscular dystrophies or sarcopenia. Currently, numerous studies exist that have employed a variety of biomaterials, cell types and strategies for maturation of skeletal muscle tissue in 2D and 3D environments. However, researchers are just at the beginning of understanding the impact of different culture settings and their biochemical (growth factors and chemical changes) and biophysical cues (mechanical properties) on myogenesis. With this review we intend to emphasize the need for new *in vitro* skeletal muscle (disease) models to better recapitulate important structural and functional aspects of muscle development. We highlight the importance of choosing appropriate system components, e.g., cell and biomaterial type, structural and mechanical matrix properties or culture format, and how understanding their interplay will enable researchers to create optimized platforms to investigate myogenesis in healthy and diseased tissue. Thus, we aim to deliver guidelines for experimental designs to allow estimation of the potential influence of the selected skeletal muscle tissue engineering setup on the myogenic outcome prior to their implementation. Moreover, we offer a workflow to facilitate identifying and selecting different analytical tools to demonstrate the successful creation of functional skeletal muscle tissue. Ultimately, a refinement of existing strategies will lead to further progression in understanding important aspects of muscle diseases, muscle aging and muscle regeneration to improve quality of life of patients and enable the establishment of new treatment options.

## Introduction

The field of regenerative medicine and tissue engineering (TE) is still one of the fastest growing research areas in biomedical science. Previous TE efforts mostly focused on tissues and organs that are associated with diseases occurring at high frequencies in 1^st^ world countries, such as the heart and the musculoskeletal apparatus with a strong emphasis on bone, cartilage, and ligaments. Muscle tissue, which for long has been relatively neglected, has gained more attention in the TE community recently. The view on muscle evolved from being the tissue mainly responsible for locomotion, thermogenesis and postural support to an endocrine organ able to secrete cytokines (termed myokines) that exert beneficial effects on surrounding tissues (Pedersen, 2011).

Tissue-specific stem cells, termed satellite cells (Beauchamp et al., 2000; Zammit et al., 2004; Yin et al., 2013; Han et al., 2016) are responsible for maintaining the regenerative capacity of skeletal muscle. Upon injury, satellite cells can re-enter the cell cycle, proliferate and either fuse to existing myofibers or generate myofibers *de novo*. Since their discovery in 1961 (Katz, 1961; Mauro, 1961), extensive research has been conducted on the regulatory mechanisms guiding satellite cell activity and their role in healthy and diseased muscle (Seale and Rudnicki, 2000; Zammit et al., 2004, 2006; Yin et al., 2013).

### Pathologic muscle states and muscle loss

Skeletal muscle TE (SMTE) aims at the functional restoration of either lost, atrophic or impaired muscle tissue. Of late, the field has particularly emphasized using cellular and acellular therapeutic approaches for pathological muscle states such as muscular dystrophies, sarcopenia, or traumatic volumetric muscle loss. In the young, regeneration generally occurs efficiently as skeletal muscle can cope with slight injuries due to its high regenerative potential. However, regeneration is inefficient when trauma causes extensive damage or when the muscle is affected by a chronic pathology. This is especially severe in the elderly, where the regenerative capacity of muscle is diminished due to a decrease in the muscle stem cell pool. This leads to progressive replacement of muscle with scar and fat tissue, causing substantial deteriorations in muscle function and motility and thus quality of life. In addition, the loss of muscle associated with aging (sarcopenia) affects a growing number of patients as the global increase in life expectancy leads to population aging. Thus there is an unmet clinical need for approaches to restore or maintain muscle function, especially in the older population which is highly affected by muscle wasting and atrophy (Chargé and Rudnicki, 2004; Ryall et al., 2008; Carosio et al., 2011; Blau et al., 2015). In contrast to sarcopenia, genetic muscle diseases, such as muscular dystrophies (MDs), result in progressive muscle weakening and breakdown starting already in childhood or middle age. MDs are a group of more than 30 rare hereditary diseases caused by mutations leading to either a dysfunction in, or lack of proteins essential for muscle stability (Theadom et al., 2014; Smith et al., 2016). MDs greatly vary in the type of muscle affected (some forms of MD may affect cardiac muscle), extent of muscle weakness, the age of onset, the rate of progression and the pattern of inheritance (Theadom et al., 2014).

Duchenne muscular dystrophy (DMD) is the most common MD affecting approximately 1 in 5,000 males (Goyenvalle et al., 2011; Mah et al., 2014; Romitti et al., 2015; Stark, 2015; Yiu and Kornberg, 2015). DMD is caused by the absence of functional dystrophin, either through deletion, point mutations, insertions or duplication. Dystrophin is a structural protein, which acts as a linker between the cytoskeleton (via the dystroglycan complex) and the surrounding extracellular matrix (ECM). Dystrophin stabilizes muscle cells under mechanical load and is essential for the maintenance of the intracellular structural organization of muscle cytoskeletal proteins in the contractile apparatus (Ervasti and Sonnemann, 2008; Constantin, 2014; Gawlik et al., 2014). Thus, lack of dystrophin predisposes muscle fibers to fragility in response to mechanical forces, leading to continuous cycles of muscle de- and regeneration (Serrano et al., 2011). Recent evidence additionally suggests that dystrophin is directly involved in regulating satellite cell behavior and that satellite cells from dystrophin knock out animals show lower proliferation rates as well as functional impairment (Sacco et al., 2010; Dumont et al., 2015b; Almada and Wagers, 2016; Dumont and Rudnicki, 2016). As a result, the muscle stem cell (satellite cell) pool is prematurely exhausted, a phenomenon somewhat analogous with aging (Webster and Blau, 1990), which eventually leads to muscle weakness, loss of motility and, in the worst case, premature death (Emery, 1993). Other MD types include Becker MD, a less severe variant of DMD, Emery-Dreifuss MD, facioscapulohumeral MD, congenital MD, limb-girdle MD or myotonic MD (Theadom et al., 2014).

To date, there is no cure for MDs. Although symptomatic treatments such as physical or drug therapies are used to delay disease progression, the prognosis for people with chronic muscle pathologies is poor. This creates a considerable world-wide socioeconomic burden for health systems, patients and caregivers alike. Sarcopenia accounts for roughly $18.5 billion per year in direct healthcare costs in the U.S. (Janssen et al., 2004; Beaudart et al., 2014). A cross-sectional study in 2014 reported the mean annual direct costs per DMD patient to range from 23,920$ to 54,270$ in Europe and the U.S., which is 7 to 16 times higher than the mean annual per capita health expenditures in these countries (Landfeldt et al., 2014). A more recent study focusing on European DMD patients and their caregivers provided similar figures but identified direct non-healthcare costs as the main part of total annual costs (Cavazza et al., 2016).

In the past, research on regenerative therapies for diseased skeletal muscle mostly focused on methods to deliver healthy myogenic cells or to restore the endogenous myogenic potential of satellite cells (Dumont et al., 2015a). Although satellite cell transplantation holds great therapeutic potential for MDs, the vast number of cells needed for treatment and their phenotypic changes after prolonged *in vitro* culture limit this approach. In addition to the restoration of the stem cell pool and host myofiber repair, healthy myogenic donor cells can also act as vectors to (re)establish expression of normal (wild-type) alleles in the muscle fibers they fuse to (Partridge et al., 1989). However, the pathomechanisms leading to MD phenotypes, muscle wasting, and atrophy are still not fully understood. In addition, the fact that some MD animal models do not faithfully recapitulate the respective disease creates another burden for translation of novel therapies into clinics. Therefore, tissue engineered *in vitro* muscle (disease) model systems can serve as an alternate pre-clinical approach to gain further insight into the molecular causes and potential treatments of chronic pathological muscle states.

### Skeletal muscle TE

**Figure 1:**
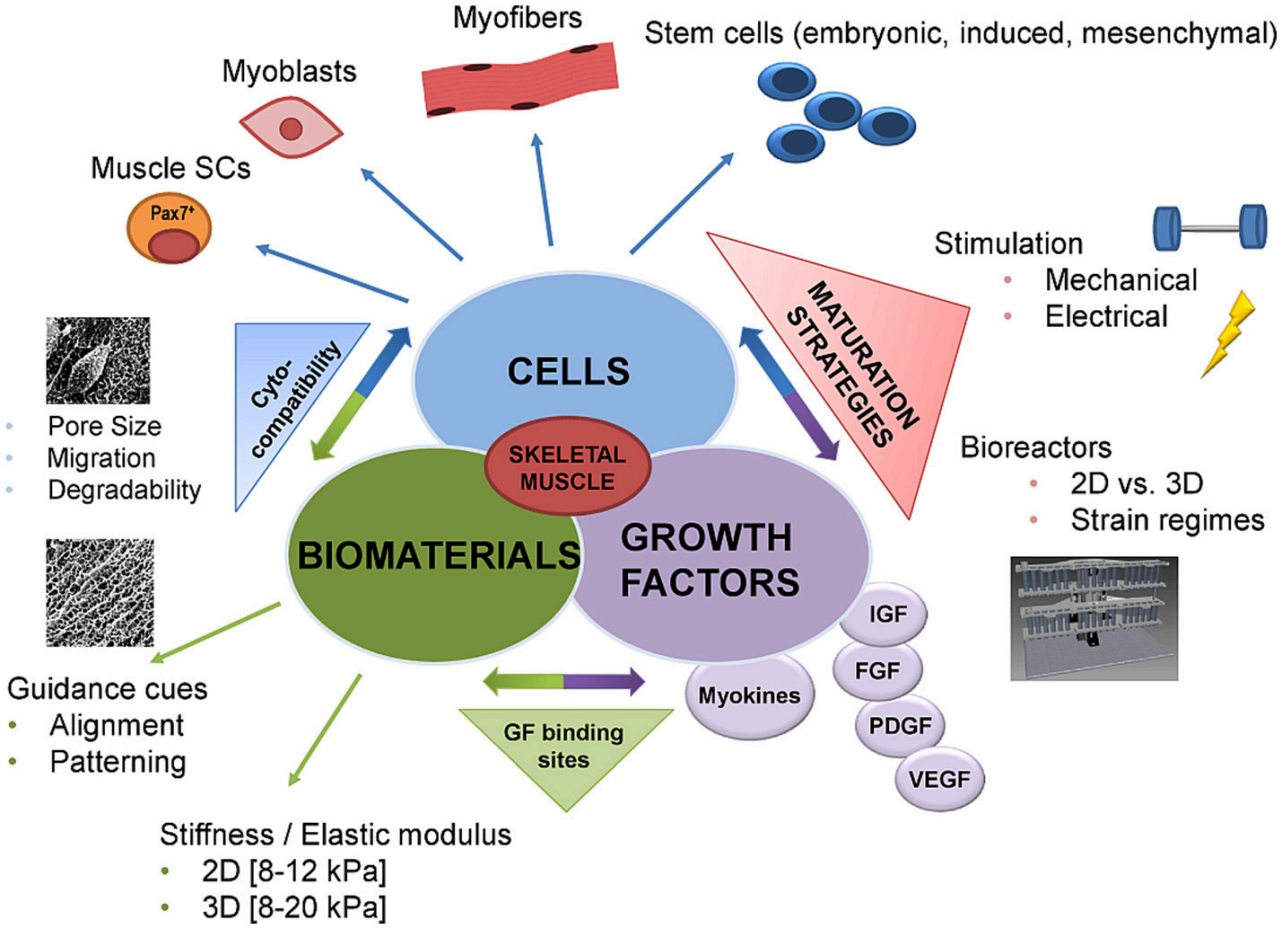
Advances in skeletal muscle tissue engineering—from classic to functional approaches. Until recently, the classic tissue engineering approach was the combination of the following components: biomaterials, cells, and growth factors. In recent years, this classic triad was combined with novel methodologies allowing for more biomimetic approaches. Advances in cross-linking chemistry made it possible to link growth factors to the biomaterial or to provide growth factor binding sites. In addition, guidance cues like patterning or alignment of the biomaterial, as well as the mechanical properties, have been demonstrated to significantly influence cell behavior such as adhesion, migration, and maturation. Likewise, the number of cell types that can potentially be used has increased ranging from cell lines and primary cells to muscle stem cells and cells with mesenchymal stem cells characteristics. One of the major advances in the past has been the incorporation of dynamic culture systems into existing SMTE approaches to improve tissue maturation. In this respect, the most commonly used techniques are electrical or mechanical stimulation via sophisticated bioreactor systems. These bioreactors allow controlled provision of different mechanical or electrical stimuli to drive both early myogenesis and functional maturation. GF, growth factor; 2D, 2-dimensional; 3D, 3-dimensional; SCs, stem cells; IGF, insulin growth factor; FGF, fibroblast growth factor; PDGF, platelet derived growth factor; VEGF, vascular endothelial growth factor.

Current clinical strategies to restore muscle function are limited to symptomatic treatments and, consequently, healthcare costs are progressively rising; e.g., healthcare costs of direct and indirect traumatic injury in the year 2000 was greater than $400 billion in the US (Corso et al., 2006). SMTE constitutes a promising tool to lower this immense socioeconomic burden, as it enables the creation of new muscle to replace lost tissue without the need of donor tissue. Furthermore, SMTE can be used to study muscle development, and the impact of biomaterials and mechanical cues on myogenesis and muscular disorders in *in vitro* (disease) models (Juhas et al., 2015). Conducting traditional studies on muscle biology in 3D settings, which more closely mimic the physiological microenvironment of the whole organ (Bursac et al., 2015), is the new state of the art in this rapidly growing field (Figure 1). However, so far, TE only successfully entered clinics when it comes to skin, bone or cartilage replacement and regeneration (Horch et al., 2000; Chang et al., 2003; Kojima et al., 2003; Kopp et al., 2004; Oakes, 2004; Vangsness et al., 2004).

Current clinical approaches to compensate for lost skeletal muscle tissue are to transfer skeletal muscle tissue from other sites of the body to the area of injury (free functional muscle transfer). However, this causes donor site morbidity and an extra surgical procedure resulting in additional stress for the patient (Qazi et al., 2015). The gold standard is the use of freestanding flaps which include functional vessels as tissue grafts. Although free functional muscle transfer is still considered the best option for restoring function in otherwise non-reconstructable muscles, a return to pre-injury levels of muscle strength and functionality does not usually occur. Thus, many research groups are now focusing on *in vitro* SMTE, providing new remarkable data for this field, some of which will be discussed in more detail in the subsequent sections (Engler et al., 2004; Huang et al., 2004; Matsumoto et al., 2007; Lam et al., 2009; van der Schaft et al., 2013; Kurth et al., 2015; Bersini et al., 2016). To date, the majority of *in vitro* SMTE strategies aim at creating functional skeletal muscle tissue in the lab to offer new therapeutic possibilities for patients suffering from volumetric muscle loss, sarcopenia or genetic muscle disorders (Law et al., 1993; Guettier-Sigrist et al., 1998). Given the current clinical treatment limitations and the rising prevalence of pathological muscle states (especially sarcopenia), these patients would greatly benefit from further research on alternative therapeutic approaches.

Another approach is *in vivo* SMTE which involves introducing cells with myogenic potential (Bach et al., 2004), either as bolus injections or in combination with a scaffold biomaterial, into the site of injury to form and regenerate new muscle tissue (McCullen et al., 2011). However, this strategy is limited by the vast amount of cells needed (Bach et al., 2004). Alternatively, the cell-free approach of *in situ* SMTE has been introduced (Jana et al., 2013; Wang et al., 2014), where instructive biomaterials are grafted into a muscle defect to trigger the endogenous regenerative potential and regenerate the diseased tissue via release of bioactive signaling molecules from the biomaterial implanted into the patient (Qazi et al., 2015). *Ex vivo* SMTE demonstrates an alternative strategy to *in vivo* approaches, where autologous cells are expanded in cell culture beforehand and eventually reintroduced into the defect site for regeneration (Barrilleaux et al., 2006; Stern et al., 2009).

With this review we would like to highlight the state of the art in SMTE, trigger ideas for refinements and provide the scientific community with putative strategies and criteria to increase the performance and maturity of tissue engineered muscle. Additionally, we give an outlook on future challenges and general considerations for SMTE applications in healthy and diseased muscle.

### Factors influencing the myogenic outcome (*in vitro* and *in vivo)*

**Figure 2:**
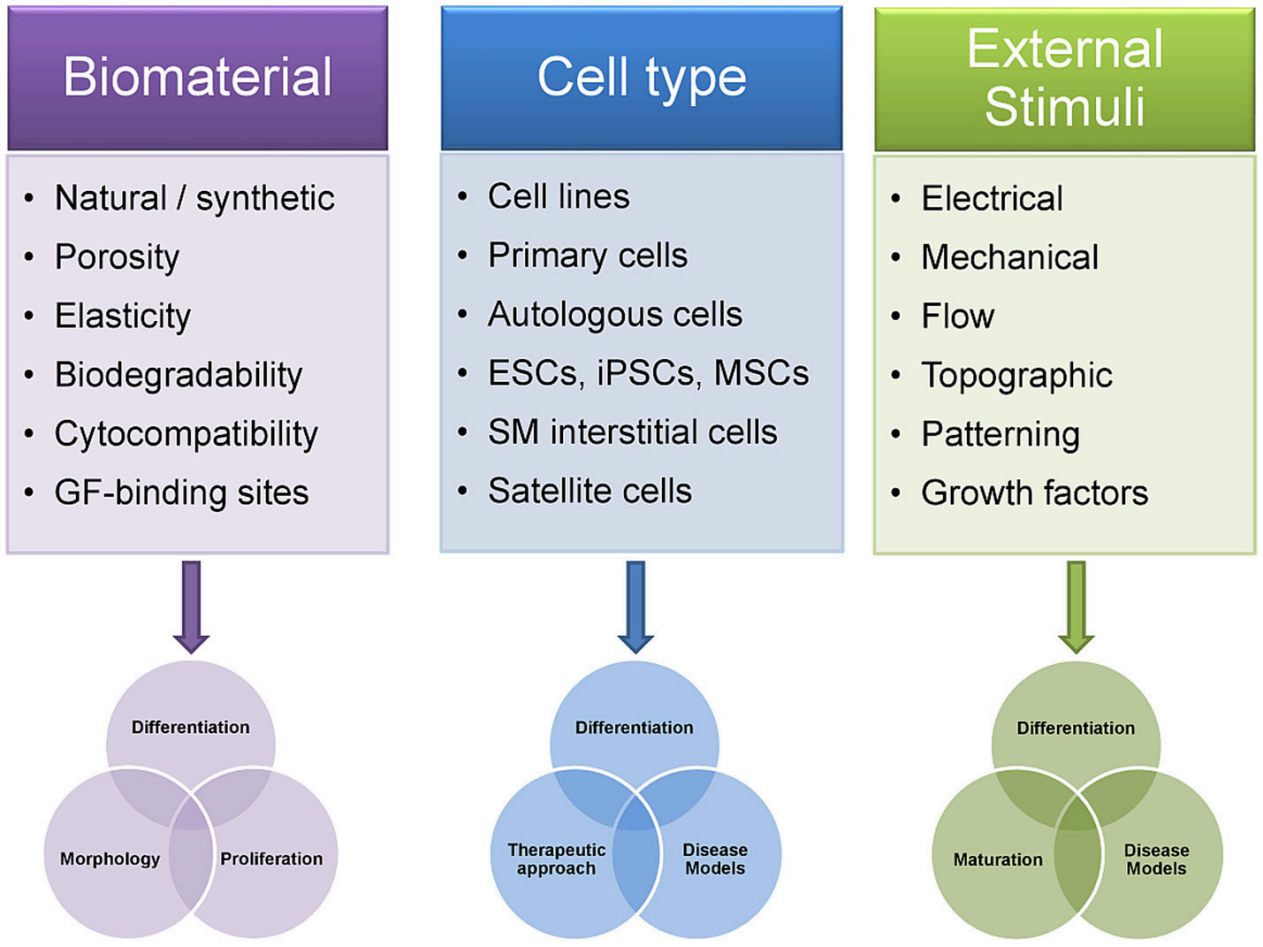
Differences in experimental design of skeletal muscle tissue engineering approaches influence outcome. The choice of the biomaterial and its biophysical properties influence the TE construct in terms of cell adhesion, migration, morphology, proliferation, and differentiation. Notably, differentiation of muscle cells into contractile myofibers is highly dependent on factors such as matrix elasticity, porosity or the availability of growth factors within the construct. The selection of the appropriate cell type is of equal importance as it partially predetermines which scientific questions can be answered using a given SMTE approach. Thus, changing cell types within the same SMTE setup can increase its application range, from studies on different stages in myogenesis or disease modeling to transplantation or cellular gene therapy. Finally, application of external stimuli to cells embedded in biomaterials greatly enhances myogenic maturation. Patterning of the biomaterial via provision of defined topographical cues can drive cell differentiation and further enables control over cell/myofiber arrangement. As engineered muscles are required to create sufficiently large contractile forces upon transplantation, the importance of dynamic culture systems using such stimulation strategies has been unambiguously shown. GF, growth factors; ESCs, embryonic stem cells; iPSCs, induced pluripotent stem cells; MSCs, mesenchymal stem cells; SM interstitial cells, skeletal muscle interstitial cells.

*In vitro* SMTE relies on efficient maturation strategies to generate functional 3D skeletal muscle constructs, which firstly requires biomaterials as scaffolds. These scaffold matrices should offer adequate physicochemical properties as well as bioactive cues like incorporated growth factors to enhance myogenic differentiation or cell adhesion motives to improve cellular attachment. Additionally, potent myogenic cells that are able to differentiate into mature myotubes under appropriate environmental conditions are a prerequisite (Bursac et al., 2015). Finally, effective stimulation strategies in the form of mechanical, electrical or electromechanical stimulation are needed to trigger cell alignment, fusion, and differentiation (Figure 2). After densely packed arrays of aligned myotubes are generated, the ultimate goal is to implement methods to (pre)vascularize and innervate such muscle constructs before they can serve as functional transplants.

### Biomaterials in SMTE

Natural biomaterials are biocompatible and biodegradable, and thus constitute favorable biomaterials for SMTE. They possess tunable mechanical and structural properties such as porosity, topographical cues, and the option of functionalization with growth factors and/or cell adhesion motives. Furthermore, natural hydrogel materials can be molded into different shapes, which is advantageous for repairing volumetric muscle defects that usually have irregular shapes. However, natural biomaterials harbor potential immunogenicity and sometimes lack of mechanical strength (ASM International, 2003; Qazi et al., 2015). The most commonly used natural biomaterials in SMTE are **collagen** (Vandenburgh et al., 1988; Shansky et al., 1997; Okano and Matsuda, 1998a,b; Powell et al., 2002; Cheema et al., 2003; Kroehne et al., 2008; Bian and Bursac, 2009; Rhim et al., 2010; Hinds et al., 2011; Ma et al., 2011; Smith et al., 2012), **fibrin** (Huang et al., 2004; Beier et al., 2006; Borschel et al., 2006; Matsumoto et al., 2007; Bian and Bursac, 2009, 2012; Lam et al., 2009; Hinds et al., 2011; Page et al., 2011; Liu et al., 2012, 2013; Heher et al., 2015), **alginate** (Shapiro and Cohen, 1997; Hill et al., 2006a,b; Borselli et al., 2010, 2011; Liu et al., 2012; Wang et al., 2014), **Matrigel**® (Grefte et al., 2012; Juhas and Bursac, 2014), **hyaluronic acid (HA)** (Wang et al., 2009; Rossi et al., 2011; Monge et al., 2012), **gelatin** (Hosseini et al., 2012; Yang et al., 2014), **silk fibroin** (Mandal and Kundu, 2009), **chitosan** (Jana et al., 2013), and **decellularized tissues** (Borschel et al., 2004; Conconi et al., 2005; De Coppi et al., 2006; Mase et al., 2010; Merritt et al., 2010; Machingal et al., 2011; Perniconi et al., 2011; DeQuach et al., 2012; Wolf et al., 2012; Corona et al., 2014; Sicari et al., 2014). Common synthetic biomaterials are manufactured from biodegradable polyesters of **polyglycolic acid**, **polyethylene glycol (PEG)**, **polycaprolactone**, **poly(lactic-co-glycolic acid)**, and **poly-l-lactic acid** (Huang et al., 2006a; Choi et al., 2008; Jun et al., 2009; Aviss et al., 2010; Kim et al., 2010a,b; Ku et al., 2012; Chen et al., 2013; Yang et al., 2014). These synthetic biomaterials are versatile in use as they are degradable (over weeks to years, depending on the formulation and degree of cross-linking), allow for precise control over their physicochemical properties (e.g., degradation rate, stiffness/elasticity or the presence of topographical or biochemical cues) and usually are considerably cheaper than natural biomaterials. Additionally, they can be used in the form of hydrogels (Grizzi et al., 1995). However, they do not always support cell attachment and adhesion, can potentially cause inflammatory responses (after degradation or through prolonged persistence at the injury site *in vivo*) and lack biomimicry of the native ECM (Kim et al., 2010b). Therefore, they are often combined or coated with natural biomaterials to present biological recognition cues e.g. integrin-binding motives like Arg-Gly-Asp to increase cell attachment (Qazi et al., 2015). An overview of commonly used biomaterials for SMTE and their advantages and disadvantages is given in Table 1.

**Table 1:** Commonly used biomaterials and their useful properties for SMTE.

**Biomaterial**	**Natural/Synthetic**	**Advantages**	**Disadvantages**	**Types of scaffolds**	**Authors**
Fibrin	Natural	Biocompatible, biodegradable, combination of materials possible, functionalization with growth factors, cell encapsulation, injectable, cell adhesive cues, tunable porosity, can enhance myoblast differentiation	Potential immunogenicity, limitation in fabrication due to denaturation, lack of mechanical strength	Hydrogels (application as 3D scaffolds), 2D pattered surfaces, coatings	ASM International, 2003; Huang et al., 2004; Borschel et al., 2006; Matsumoto et al., 2007; Bian and Bursac, 2009, 2012; Lam et al., 2009; Liu et al., 2013; Heher et al., 2015; Qazi et al., 2015
Collagen	Natural	Biocompatible, biodegradable, combination of materials possible, interconnectivity, macroporous structure, topographical cues, cell adhesive cues, tunable porosity, can enhance myoblast differentiation, injectable	Potential immunogenicity, limitation in fabrication due to denaturation, lack of mechanical strength	Hydrogels (application as 3D scaffolds), 2D pattered surfaces, coatings	Vandenburgh et al., 1988; Shansky et al., 1997; Okano and Matsuda, 1998a,b; Powell et al., 2002; ASM International, 2003; Cheema et al., 2003; Kroehne et al., 2008; Bian and Bursac, 2009; Rhim et al., 2010; Hinds et al., 2011; Ma et al., 2011; Smith et al., 2012; Qazi et al., 2015; Han et al., 2016
Gelatin	Natural	Biocompatible, biodegradable, combination of materials possible, topographical cues, can enhance myoblast differentiation	Potential immunogenicity, limitation in fabrication due to denaturation, lack of mechanical strength	Coatings	ASM International, 2003; Hosseini et al., 2012; Yang et al., 2014; Qazi et al., 2015
Alginate	Natural	Biocompatible, biodegradable, high surface area, interconnectivity, functionalization with growth factors, cell encapsulation, injectable, cell adhesive cues, tunable porosity, macroporous structure, minimally invasive	Potential immunogenicity, limitation in fabrication due to denaturation, lack of mechanical strength, need for adhesive cues (RGD)	Hydrogels (application as 3D scaffolds)	ASM International, 2003; Hill et al., 2006a,b; Borselli et al., 2011; Liu et al., 2012; Wang et al., 2014; Qazi et al., 2015; Han et al., 2016
Chitin/chitosan	Natural	Biocompatible, biodegradable, topographical cues, varying mechanical properties, tunable porosity	Potential immunogenicity, limitation in fabrication due to denaturation, lack of mechanical strength	Grooved scaffolds, application as 3D scaffolds	ASM International, 2003; Jana et al., 2013; Qazi et al., 2015
Decellularized tissues/ECM	Natural	Tunable structural integrity, native structural and biochemical cues, matches host tissue, mechanical properties, bioactive	Potential immunogenicity, processing relies on chemical/biological agents which break down natural ECM structure, acquisition of material more complicated - especially for human tissue	Hydrogels (application as 3D scaffolds), full thickness *in vitro*	ASM International, 2003; Borschel et al., 2004; Conconi et al., 2005; De Coppi et al., 2006; Mase et al., 2010; Merritt et al., 2010; Machingal et al., 2011; Perniconi et al., 2011; DeQuach et al., 2012; Wolf et al., 2012; Corona et al., 2014; Sicari et al., 2014; Qazi et al., 2015
Hyaluronic acid	Natural	Biocompatible, biodegradable, tunability, injectable, cell encapsulation, minimally invasive	Potential immunogenicity, limitation in fabrication due to denaturation	Hydrogels (application as 3D scaffolds)	ASM International, 2003; Rossi et al., 2011; Wang et al., 2014; Qazi et al., 2015; Han et al., 2016
PEG	Synthetic	Biocompatible, high tunability, injectable, cell encapsulation, minimally invasive	Recellularization is slow, poor support in remodeling, lack of adhesive sites for cell attachment	Hydrogels (application as 3D scaffolds)	Kim et al., 2010b; Bao Ha et al., 2013; Qazi et al., 2015; Han et al., 2016
PLLA	Synthetic	Biocompatible, combination of materials possible, offer topographical cues, tunability (e.g., groove width and depth), electrically conductive, can enhance myoblast differentiation	Recellularization is slow, poor support in remodeling, lack of adhesive sites for cell attachment	2D patterned surfaces, electrospun fibers with tunable ridge width, alignment and variable composition of polymer material	Gunatillake et al., 2003; Huang et al., 2006a; Bao Ha et al., 2013; Qazi et al., 2015
PLGA	Synthetic	Biocompatible, biodegradable, combination of materials possible, offer topographical cues, tunability (e.g., groove width and depth), electrically conductive, can enhance myoblast differentiation	Recellularization is slow, poor support in remodeling, lack of adhesive sites for cell attachment	2D patterned surfaces, electrospun fibers with tunable ridge width, alignment and variable composition of polymer material	Gunatillake et al., 2003; Aviss et al., 2010; Bao Ha et al., 2013; Yang et al., 2014; Qazi et al., 2015
PCL	Synthetic	Biocompatible, biodegradable, combination of materials possible, offer topographical cues, tunability (e.g., groove width and depth), electrically conductive, can enhance myoblast differentiation, can be used in drug delivery systems	Recellularization is slow, poor support in remodeling, lack of adhesive sites for cell attachment	2D patterned surfaces electrospun fibers with tunable ridge width, alignment and variable composition of polymer material	Gunatillake et al., 2003; Choi et al., 2008; Kim et al., 2010b; Ku et al., 2012; Bao Ha et al., 2013; Chen et al., 2013; Qazi et al., 2015

#### Hydrogels

Hydrogels are particularly popular in SMTE due to their tunability regarding structure, shape and mechanical stability as well as their amenability to incorporate contact guidance and biochemical cues. Additionally, hydrogels can be functionalized with growth factors or other bioactive molecules to enhance regeneration (Hill et al., 2006a; Ostrovidov et al., 2014; Qazi et al., 2015). 3D hydrogels promote a spatially uniform cell distribution after encapsulation, enabling the generation of dense tissue constructs through high initial cell seeding densities and hydrogel compaction by the cells over time. The high amount of cell-cell contacts promotes and enhances myogenic fusion and increases myofiber length and thickness (Heher et al., 2015). Furthermore, 3D environments mimic the physiological conditions of the tissue more closely than 2D cultures. The use of hydrogel-based biomaterials is a promising strategy to introduce therapeutic myogenic precursor cells into a defect for subsequent formation of new muscle tissue *in vivo* (Han et al., 2016). Notably, hydrogels can be injected in a minimally invasive manner to support or fill void spaces after muscle trauma or disease (Qazi et al., 2015).

Collagen is the most abundant protein in the human body and the main constituent of natural ECM, which is why it has been used in a multitude of TE applications (Lee et al., 2001). However, if muscle satellite cells (MuSCs) are used, laminin has to be added to match the specific integrin complex formed by α7 and β1 isoforms (Blanco-Bose et al., 2001). In a pioneering study, Vandenburgh et al used collagen gels to incorporate and differentiate avian myoblasts into contractile myotubes with structural characteristics similar to neonatal myofibers (Vandenburgh et al., 1988). Since then, many other groups have used myogenic precursor cells combined with collagen hydrogels (Cheema et al., 2003; Rhim et al., 2007; Ma et al., 2011; Smith et al., 2012). Okano et al. highlighted that C2C12 myoblasts combined with 3D collagen gels led to differentiation into multinucleated aligned myotubes, successful capillary infiltration *in vivo*, and remodeling after implantation (Okano and Matsuda, 1998b).

Another natural biomaterial for hydrogel production is alginate, a polysaccharide found in seaweed, rendering it a feasible and cheap hydrogel source (Boontheekul et al., 2005; Andrejecsk et al., 2013). An advantage of alginate hydrogels is the possibility to modify them, for example by introducing cell adhesive ligands or adjusting stiffness and degradability (Shapiro and Cohen, 1997; Hill et al., 2006a,b; Borselli et al., 2010; Liu et al., 2012). Alginate hydrogels are used in many medical applications, including wound healing management or the delivery of bioactive molecules due to their low toxicity and good biocompatibility (Lee and Mooney, 2012).

The ECM component HA is also used for the fabrication of hydrogels by photo cross-linking via UV light treatment (Han et al., 2016) or by chemical cross-linking (Luo et al., 2000; Collinsworth et al., 2002; Zhang et al., 2016). HA enhances myoblast proliferation and differentiation. However, degradation by hyaluronidases *in vivo* is difficult to control, which may lead to apoptosis of the introduced cells due to loss of attachment to the material (Han et al., 2016).

Fibrin is a favored biomaterial to produce hydrogels. It is the end-product of the blood clotting cascade, formed when fibrinogen is cleaved by thrombin (Helgerson et al., 2004). As fibrin is a natural component of the human body like collagen and HA, it provides attractive features, including biocompatibility, biodegradability and non-toxicity. Encapsulating myogenic cells in fibrin hydrogels provides cues to trigger growth and differentiation into myotubes and eventually to myofibers (Juhas et al., 2015). Further advantageous features include tunability of its structural network, modifiable polymerization (Han et al., 2016) and the potential for incorporating growth factors (Ahmed et al., 2008). Some studies claim fibrin to be superior to other biomaterials (e.g., collagen I) due to the strong integrin binding (integrin α7 and α5) of myotubes to fibrin (Morishima-Kawashima et al., 1995; Papers and Mayer, 2003). This effect is more pronounced in fibrin, as myotubes do not have the collagen I specific integrin α2 receptor. Therefore, a fibrin environment is more conducive to distributing contractile forces from myocytes (Juhas et al., 2015). The major drawback of fibrin is finding an appropriate material density that balances the required material integrity to mimic natural stiffness and sufficient porosity for nutrient transport and cell migration (Helgerson et al., 2004; Brown and Barker, 2014). Fibrin hydrogels have been used successfully in numerous SMTE approaches using different stimuli to enhance differentiation (Huang et al., 2004; Borschel et al., 2006; Matsumoto et al., 2007; Lam et al., 2009; Liu et al., 2013; Heher et al., 2015).

Injectable hydrogels derived from decellularized muscle ECM may offer a more flexible approach than whole decellularized muscles (DeQuach et al., 2012). Although muscle ECM breakdown and subsequent processing into hydrogels destroys all existing architectural cues of the ECM (such as the vascular bed), the hydrogel formulation's composition in terms of proteins, growth factors and cytokines is preserved and can still instruct endogenous regenerative processes. Importantly, hydrogels can be produced from xenogeneic ECM sources, such as porcine dermis, submucosa or urinary bladder (Wolf et al., 2012; Badylak et al., 2016), circumventing the need for autologous muscle ECM which would be inapplicable in clinics due to donor site morbidity.

Synthetic hydrogels based on PEG are cytocompatible and offer tremendous variability for chemical manipulation. For SMTE applications, PEG can be tailored to mimic the natural stiffness of skeletal muscle tissue (Juhas et al., 2015) and seems to be a promising biomaterial for myogenic differentiation (Han et al., 2016). Laminin-coated PEG hydrogels, as an example of combined synthetic and natural materials, favored MuSC proliferation and differentiation *in vitro* (Han et al., 2016). PEG combined with fibrinogen constitutes a promising scaffold to embed skeletal muscle-derived pericytes (Fuoco et al., 2014) or mesoangioblasts (Fuoco et al., 2012, 2015) and favors differentiation of cells and regeneration of muscle tissue. Moreover, PEG-based hydrogels can be functionalized with different growth factors to directly promote muscle regeneration *in situ*, recruit endogenous stem cells to the site of injury, or enhance differentiation of muscle progenitor cells on/in the gel (Hammers et al., 2012; Rybalko et al., 2015).

### Cells for muscle tissue engineering

Another essential factor influencing the myogenic outcome is choosing appropriate cells when generating functional muscle tissue constructs. The pool of cell types scientists can choose from has grown enormously in recent years and a variety of cell populations that are able to differentiate along the myogenic lineage have been identified (Fishman et al., 2013). In addition, new techniques, such as the generation of patient-specific induced pluripotent stem cells (iPSCs) or gene editing via the CRISPR/Cas9 technology have opened new therapeutic possibilities, especially for the treatment of MDs.

The two main groups of cells potentially being used for SMTE are either freshly isolated and expanded primary cells or immortalized cell lines. The main application of immortalized cells is the establishment of model systems, whereas primary cells are used in clinical applications and for implant studies. Myoblasts, satellite cells and stem cells from various sources are employed in different therapeutic approaches to improve muscle regeneration and function (Bach et al., 2004). The most prominent type of primary myogenic cells are MuSCs, which demonstrate a high proliferative capacity, have the ability to self-renew and differentiate into myotubes (Zammit et al., 2004; Qazi et al., 2015). Autologous MuSCs cultured with homologous acellular muscular matrices enhances their engraftment, and subsequently those matrices can be used as transplants to compensate for tissue loss (Marzaro et al., 2002). A drawback is that they have poor survival and engraftment rates after injection into damaged tissue (Mouly et al., 2005). MuSCs can be isolated either via enzymatic digestion of muscle tissue or via cellular outgrowth by plating single muscle fibers onto protein-coated dishes, which serve as a niche for satellite cells (Zammit et al., 2006; Juhas et al., 2015). However, a drawback of satellite cells is that once activated and differentiated into myotubes they cannot be brought back to a self-renewing state. Thus, the pool of cells able to proliferate and build new myotubes is eventually exhausted (Shadrin et al., 2016).

Over the years, other tissue resident cells have been discovered, namely interstitial skeletal muscle progenitor cells, which constitute a heterogeneous cell pool and seem to derive from the interstitium near the blood vessels (Shadrin et al., 2016). They offer a great regenerative potential and have already been used in studies of rodent and human SMTE. Pw1 interstitial cells, a fraction of interstitial skeletal muscle progenitor cells (Relaix et al., 1996), originate upstream of MuSCs in the muscle precursor lineage and can induce the formation of MuSCs. Therefore, their presence is a key factor in the satellite cell niche (Malecova and Puri, 2012). In the murine model, Pw1 interstitial cells enhanced muscle regeneration by releasing paracrine growth factors. Other subsets of skeletal muscle interstitial cells that play important roles in inducing muscle differentiation are fibroadipogenic progenitors, pericytes, and mesoangioblasts. All three cell populations are promising for SMTE approaches, since they are capable of ameliorating myogenic regeneration, offer high proliferative rates, and can be genetically modified (Minasi et al., 2002; Dellavalle et al., 2007; Tonlorenzi et al., 2007; Crisan et al., 2008; Joe et al., 2010; Birbrair et al., 2013; Ostrovidov et al., 2015). These characteristics also increase their relevance for potential MD treatments (Sampaolesi et al., 2003, 2006; Tedesco and Cossu, 2012; Meregalli et al., 2013). Furthermore, these cells are suitable for regenerative medicine approaches due to their good survival rates and their ability to fuse to preexisting myofibers, thereby promoting muscle regeneration *in vivo* (De Angelis et al., 1999; Minasi et al., 2002; Dellavalle et al., 2007; Tonlorenzi et al., 2007; Joe et al., 2010; Tedesco and Cossu, 2012).

Another cell type with great potential for regenerative medicine is mesenchymal stem cells (MSCs). MSCs are multipotent cells capable of migrating to the site of injury to promote tissue repair (Ferrari et al., 1998; Dezawa et al., 2005) and reducing inflammation (Sassoli et al., 2012). MSCs are able to differentiate into the myogenic lineage (Dezawa et al., 2005). Furthermore, they enhance muscle fiber formation and regeneration *in vivo* (Ferrari et al., 1998; De Bari et al., 2003; Koponen et al., 2007; Lee et al., 2011; Sassoli et al., 2012). This might be due to their support of functional satellite cells when implanted in murine muscle tissue (De Bari et al., 2003) and through recovery of expressed mechano growth factor, which is crucial for skeletal muscle maintenance and repair (Goldspink, 1999). This positive effect on muscle regeneration has been validated in *in vivo* disease models, where autologous MSCs were transplanted into crush trauma injuries in rats (von Roth et al., 2012; Qazi et al., 2015). MSCs' therapeutic effects may also stem from their ability to secrete soluble paracrine factors (Gnecchi et al., 2008; Sassoli et al., 2012) including Interleukin (IL)-6, IL-10, stromal cell-derived factor (Gnecchi et al., 2005; Kortesidis et al., 2005; Zhang et al., 2007; Yin et al., 2011), vascular endothelial growth factor, fibroblast growth factor (FGF), IL-1, matrix metalloproteinases (MMPs), platelet derived growth factor, transforming growth factor ß, angiopoyetin (Kinnaird et al., 2004), hepatocyte growth factor, and adrenomedullin (Ohnishi et al., 2007; Wynn, 2008; Tang et al., 2010). Via secretion of these factors, MSCs assert substantial anti-inflammatory effects by modulating the immune response (Le Blanc and Mougiakakos, 2012). However, a study by Ferrari et al reported that bone-marrow transplantation did not ameliorate the dystrophic phenotype in *mdx* mice, a widely used mouse model for DMD (Ferrari et al., 2001). One suggested reason for the low regenerative potential of MSCs in this setting was that a vast number of cell types is present in the bone-marrow, which resulted in relatively low numbers of MSCs actually being transplanted in the course of a bone-marrow transplantation (reviewed by Forcales, 2015). Alternative cells used for SMTE are L6 rat myoblasts, neonatal muscle-derived progenitor cells and xenogeneic cells derived from adult muscles from other species (van der Schaft et al., 2013).

Human or murine embryonic stem cells represent another regularly used source for obtaining skeletal myoblasts. It is possible to obtain CD73^+^ multipotent mesenchymal precursors, which can be differentiated into myoblasts by co-culturing them with C2C12 cells (Barberi et al., 2005). Since their generation by Yamanaka and Takahashi (2006), iPSCs have been widely implemented in different research areas. This technique makes it possible to reprogram cells directly from patients for autologous cell therapy of MDs (Meregalli et al., 2014; Quattrocelli et al., 2015). Since iPSCs can be derived from healthy or diseased patients, they offer great potential in TE for disease modeling and drug testing (Wobma and Vunjak-Novakovic, 2016). Such autologous patient-derived cells are non-immunogenic and, in addition, genetic defects can be corrected during *ex vivo* culture using tools such as CRISPR/Cas9. Interestingly, iPSCs generated from mesoangioblasts were shown to fuse to existing muscle with higher efficiency than iPSCs generated from fibroblasts (Quattrocelli et al., 2015). An important proof-of-concept study was performed by Tedesco et al., who used genetically corrected iPSCs derived from myoblasts or fibroblasts of limb-girdle MD patients, differentiated them into mesoangioblasts and grafted them into affected muscles in a humanized limb-girdle MD mouse model (Tedesco et al., 2012). This not only ameliorated the dystrophic phenotype and restored the depleted satellite cell pool, but importantly also demonstrated that treatment with patient-specific iPSC-derived cells can be utilized for stem cell therapy in MDs. However, it has to be noted that there are still limitations regarding both the use of embryonic stem cells, which raise ethical concerns (An and Li, 2014), and iPSCs, which entail the risk of genetic recombination and tumor formation. To date, iPSC-based regenerative stem cell therapies have not entered clinics due to these safety considerations (Lee et al., 2009; Cittadella Vigodarzere and Mantero, 2014).

Finally, one of the most widely used cell line in SMTE are C2C12 murine myoblasts, established in 1977 from MuSCs derived from a C3H mouse (Yaffe and Saxel, 1977). Many researchers start their initial experiments with these cells, as they are easy to cultivate, proliferate rapidly, and differentiate well upon serum deprivation. Thus, they represent an ideal tool to evaluate new biomaterials or bioreactor systems for the generation of skeletal muscle tissue. However, due to the immortalization of the cells, translation into clinical use is not feasible. Human cell lines, however, may still serve as attractive cells for *in vitro* studies. A recent transcriptomics analysis revealed that immortalization of C25 human myoblasts neither interferes with their myogenic potential, nor with any other aspect of cell physiology—apart from the elicited protection against senescence (Thorley et al., 2016). Biomimetic *in vitro* skeletal muscle disease models employing patient-derived human myoblast lines may therefore provide a higher predictive capability than rodent *in vivo* models.

### Stimulation strategies for enhancing maturation of 3D bioengineered muscle constructs

Besides choosing the appropriate biomaterial and cell type, another key element that needs to be addressed is suitable stimulation strategies (either mechanical-, electrical-, or electromechanical-stimulation), which are indispensable for enhanced muscle maturation *in vitro*. Cells are highly responsive to their microenvironment such as the surrounding ECM, mechanical forces, and biochemical signals. Furthermore, the mechanical properties of biomaterials, such as the material stiffness or the presence of distinct microarchitectural features, can influence cellular behavior tremendously (Engler et al., 2006, 2007; Cittadella Vigodarzere and Mantero, 2014). The stiffness/elasticity of a material is usually assessed by measuring the Young's modulus (elastic modulus) which is determined by a material's composition and capability for deformation.

One strategy to mimic the natural environment is the application of biochemical and/or biophysical stimulation to engineered constructs. Exercise can be simulated by the application of mechanical stimuli, such as cyclic and/or static strain. Exercise leads to the activation of satellite cells and subsequent fusion to already existing myofibers *in vitro* (Tatsumi et al., 2001) through triggering the release of hepatocyte growth factor and nitric oxide (NO) radicals, which in turn activate the satellite cells. NO is produced by nitric oxide synthases which are up-regulated by exercised or injured muscle tissue *in vitro* and *in vivo* (Tatsumi, 2010).

Regarding myogenesis, passive (e.g., bone elongation during development) as well as active (e.g., exercising during sport) mechanical stretching is essential for the development of skeletal muscle from embryonic to adult tissue (Goldspink et al., 1992; Heher et al., 2015). An appropriate stimulation protocol can exert a positive effect on gene regulation, protein expression and thus proliferation and differentiation of cells (Goldspink et al., 1992; Powell et al., 2002; Goldspink, 2003). Furthermore, exercise training improves fusion and alignment of myofibers (Vandenburgh and Karlisch, 1989; Corona et al., 2012; Heher et al., 2015), and enhances the generation of mature myofibers (Goldspink, 2003). Morphologically, mature skeletal muscle tissue is characterized by widespread sarcomeric patterning, which is indispensable for contraction. Moreover, mechanical stimulation causes an increase in the cross-striations of the tissue and a switch of myosin heavy chain isoforms from embryonic to adult (Juhas et al., 2015).

One of the first studies implementing mechanical stimulation was conducted by Goldberg et al. in which hypertrophy was induced by overloading of synergistic muscle within just 24 h (Goldberg, 1967; Armstrong et al., 1979). Further sophisticated mechanical stimulation protocols were conducted using bioreactors with mechanical stimuli to create dynamic 2D or 3D culture systems. These studies are listed in more detail in Table 2 (Vandenburgh and Karlisch, 1989; Okano and Matsuda, 1998b; Powell et al., 2002; Auluck et al., 2005; Cheema et al., 2005; Matsumoto et al., 2007; Liao et al., 2008; Moon et al., 2008; Candiani et al., 2010; Machingal et al., 2011; Corona et al., 2012; Smith et al., 2012; Heher et al., 2015; Qazi et al., 2015).

**Table 2:** Summary of bioreactor systems with corresponding mechanical stimulation protocols.

**Cells**	**Biomaterial**	**Set-up**	**2D/3D culture**	**Stress regime, frequency [Hz]**	**Strain [%]**	**Time span of stimulation**	**Outcome**	**Authors**
**MECHANICAL STIMULATION**								
Embryonic avian pectoralis muscle cells	Collagen constructs	Mechanical cell stimulator device (computerized)	3D	Cyclic ramp stretch at a rate of 0.35 mm/h	Up to 300%	3 days	Increase of proliferation, fusion and myotube length	Vandenburgh and Karlisch, 1989
C2C12 myoblasts	Collagen hydrogel	Stimulation of rod-shaped tissue via custom-designed stress chamber	3D	Cyclic, 60 Hz	5%	7 days	Dense, oriented myotubes	Okano and Matsuda, 1998b
Myoblasts	Porous collagen scaffold	Stimulation via bio-Stretch system	3D	Continuous or cyclic uniaxial rapid ramp stretch (RRS) or cyclical ramp strain (CRS)	7.5 and 15%	6 h	MMP-2 expression, and hence extracellular matrix remodeling, is up-regulated in response to strain	Auluck et al., 2005
Human skeletal muscle cells	Collagen/Matrigel® Mix	Stimulation of hBAMs by custom-made mechanical cell stimulator	3D	5-pulse at 5 Hz bursts for 2 min afterwards 28 min resting phase	5% on day 8–10, 10% on day 10–12, 15% on day 12–16	8 days	Enhance myofiber diameter and area diminished tissue stiffness	Powell et al., 2002
Myoblasts	Fibrin	Use of custom-made device	3D	Tensile strain	25 or 50%	7 days	Fiber alignment along direction of strain	Matsumoto et al., 2007
Primary muscle precursor cells	Collagen based acellular ECM scaffolds	Computerized bioreactor system	3D	Cyclic stretch	10%	5–21 days	Generation of fast twitch and tetanic force after implantation	Moon et al., 2008
Adult rat Muscle progenitor cells (MPCs)	BAM scaffolds from acellular bladder ECM	Stimulation via computer-controlled bioreactor system	3D	Cyclic stretch 3x per min, for the first 5 mins every hour	10%	7 days	After implanting improved host recovery	Machingal et al., 2011; Corona et al., 2012
Rat primary cells	Collagen	Sliding chamber model	2D	Isometric tension	n/a	21 days	3D constructs made of aligned myotubes	Smith et al., 2012
C2C12 myoblasts	Collagen constructs	Mechanical loads applied by tensioning culture force monitor bioreactor	3D	Repetitive cyclic stretch Ramp stretch	1% 10%	up to 12 h	IGF-IEa > upregulated by single ramp stretch, reduced by repetitive cyclic stretch MGF > upregulated by single ramp stretch and cyclic stimulation	Cheema et al., 2005
C2C12 myoblasts	Aligned electrospun polyurethane (PU) fibers	Tubular custom-made setup, computer program controlled	2D/3D	Repetitive cyclic stretch, 1 Hz for 1 h every 6 h	5 or 10% with or without pre-strain of 5% static	2–14 days	Alignment, contractile proteins	Liao et al., 2008
C2C12 myoblasts	Biodegradable microfibrous scaffold [DegraPol(R)]	Stimulation of constructs via custom made bioreactor	3D	2 days ramp stretch (3.3%), afterwards cyclic stretch (5 pulse, 0.5 Hz, 3.4% burst stretches	6.7%	7–10 days	Enhanced MHC expression	Candiani et al., 2010
C2C12 myoblasts	Fibrin hydrogels	Stimulation of constructs via MagneTissue bioreactor	3D	Static strain	10%	9 days	Increased gene expression, myotube diameter and length	Heher et al., 2015

Muscle tissue can also be stimulated with electrical stimulation, which positively affects myogenic gene regulation as well as protein expression (Goldspink et al., 1992; Powell et al., 2002; Goldspink, 2003). Motor neurons are responsible for innervating muscle fibers and the signal inducing contraction of the muscle tissue is distributed via branched axons (Purves et al., 2001). Electrical stimulation aims to recapitulate the processes of innervation by fast and slow motor neurons, which are responsible for the switch of muscle fiber types (Wehrle et al., 1994; Khodabukus et al., 2015). Electrical stimulation of mouse myoblasts improves myogenic differentiation (Park et al., 2008) and enhances their contractile properties compared to unstimulated controls (Salmons et al., 2005; Fujita et al., 2007). In monolayer myogenic cultures, twitches happen spontaneously after the formation of myotubes, but electrical stimulation is needed for a controlled and sustained contraction. Chronic periods of electrical stimulation are relevant for the formation of mature phenotypes in muscle tissue constructs as well as to improve their contractile properties (Kasper et al., 2017). Many groups have applied sophisticated electrical stimulation protocols to muscle cells *in vitro* (Stern-Straeter et al., 2005; Huang et al., 2006b; Fujita et al., 2007; Donnelly et al., 2010; Langelaan et al., 2011; Khodabukus and Baar, 2012) (Table 3).

**Table 3:** Summary of bioreactor systems with corresponding electrical and electro-mechanical stimulation protocols.

**Cells**	**Biomaterial**	**Set-up**	**2D/3D culture**	**Stress regime, frequency (Hz), (V), pulse width, strain (%)**	**Time span of stimulation**	**Outcome**	**Authors**
**ELECTRICAL STIMULATION**							
Primary rat myoblasts	Fibrin	Biphasic stimulation of culture slide chamber via platinum electrodes	3D	6.8 mA (4 ms duration), bursts at 250 ms > intervals every 4 s	Up to 8 days	No evidence of differentiation and fusion	Stern-Straeter et al., 2005
Rat primary cells (fast muscle)	Fibrin	Stimulation of myooid constructs via custom build force transducer	3D	5 pulses at 20 Hz/4 s at 5 V, 1.5 ms	After culturing of 14 days	Increase in contractility and an enhancement of 15% in TPT and 14% in $\frac{1}{2}$ RT	Huang et al., 2006b
90% C2C12, 10% 3T3	Fibrin	Stimulation of myooid constructs via custom-made stimulation bioreactor	3D	4 pulses, periods at 1.25, 2.5 and 5 V/mm, 0.1 ms in a 400 ms train, recovery of 3.6 s	7 days	2.5 V/mm seemed to be the optimum as it demonstrated a stronger force production and excitability	Donnelly et al., 2010
90% C2C12, 10% 3T3	Fibrin	Stimulation of constructs via custom-made electrical stimulator	2D/3D	0.7, 1, 1.4 V/mm, 0.25 to 1, 4, 9, and 16 ms pulse width	24 h	Electrical field higher than 0.7–2.5 V/mm + pulse width of 1–4 ms > showed enhanced force productions, stronger force dynamics	Khodabukus and Baar, 2012
C2C12	n/a	Electrical pulse stimulation of coverslips	2D	40 V/60 mm, 1 Hz	8 days after differentiation for 1, 2, or 6 h	Development of contractile activity by application of 2 h stimulation at 1 Hz, decrease of contractility when applying electrical stimulus for more than 4 h	Fujita et al., 2007
C2C12, muscle progenitor cells (MPCs)	Collagen type I	Bipolar field stimulation of mBAMs via C-Pace Culture Pacer	2D/3D	4 V/cm, 6 ms pulses at 2 Hz	48 h started on day 0, 1, 2, or 3	More mature cross-striations in MPC mBAMs than C2C12 and fast to slow MHC isoform switch in MPC mBAMs	Langelaan et al., 2011
**ELECTROMECHANICAL STIMULATION**							
**Cells**	**Biomaterial**	**Set-up**	**2D/3D Culture**	**Stress regime, pulse width, frequency (Hz), Strain (%)**	**Time span of simulation**	**Outcome**	**Authors**
C2C12 myoblasts	Electro spun polyurethane (PU)	Tubular custom-made set up, computer program controlled	2D/3D	Cyclic stretch, 4 V/mm, 1 Hz, 5%	1 h mechanical strain, resting time 5 h + 7 days of electrical stimuli	Enhanced myotube formation, increase in alpha actinin + MHC	Liao et al., 2008

To the best of our knowledge, so far there is only one published study combining both electro-and mechanical stimulation for engineering mature muscle constructs (Liao et al., 2008) (Table 3). In literature, there is only one bioreactor system reported, which combines the application of electrical and mechanical stimulation of 3D constructs. It is a commercially available system from EBERS Medical Technology, Spain, and allows for media perfusion under sterile conditions (Kasper et al., 2017). An overview of bioreactor systems used in SMTE with their used electrical stimulation protocols and the observed outcome is given in Table 3.

### Myokines released by exercised muscle tissue and their effect on various tissue types

Myokines are another factor influencing muscle as well as other tissues and therefore might offer an interesting therapeutic option to treat patients in future. They are released by muscle tissue in response to exercise training. It is known that regular exercise has beneficial effects on overall health status. Accumulating epidemiologic evidence suggests that physical activity plays an independent role in preventing frequent chronic diseases like osteoporosis, diabetes, Alzheimer's, osteoarthritis or degenerative muscle conditions, and that the beneficial effects of exercise training are partially due to secreted myokines (Dunstan, 2011; Pedersen, 2011; Egan and Zierath, 2013).

Skeletal muscle has been recognized as an endocrine organ due to its ability to produce, store and secrete hormones and myokines. In particular, myokines are able to affect and regulate inflammatory and metabolic processes in muscle and in many other tissues in an endocrine or paracrine manner (Pedersen and Febbraio, 2008; Pedersen, 2009; Girgis et al., 2014; Tagliaferri et al., 2015). To date, there are 69 putative myokines, which are released via exercise training (Catoire et al., 2014).

So far, the most prominently investigated myokines are IL-6 (Pedersen, 2009), IL-7 (Haugen et al., 2010), IL-8 (Nielsen and Pedersen, 2007), IL-15 (Quinn et al., 2008), leukemia inhibitory factor (Broholm et al., 2011), FGF-21 (Izumiya et al., 2008; Hojman et al., 2009), insulin-like 6 (Zeng et al., 2010), follistatin-like 1 (Ouchi et al., 2008), musculin (Nishizawa et al., 2004), irisin (Boström et al., 2012), myonectin (Seldin et al., 2012), secreted protein acidic rich in cysteine (SPARC) (Songsorn et al., 2016), and Meteorin-like 1 (Rao et al., 2014).

IL-6 is a pleiotropic myokine and acts on muscle tissue by influencing satellite cell activation and differentiation, which is usually triggered by stress due to injury or mechanical stimulation (Muñoz-Cánoves et al., 2013). It is the first myokine to be released after acute exercise (Agarwal, 2017). Besides primarily acting on muscle, other organs such as adipose tissue, the liver and the brain are responsive to secreted IL-6 (Pedersen et al., 2001). Secondly, it negatively regulates pro-inflammatory cytokines (Pedersen and Febbraio, 2008; Benatti and Pedersen, 2014) such as tumor necrosis factor alpha and elevates levels of anti-inflammatory cytokines e.g., IL-10 and IL-1 receptor antagonist released from leukocytes (Pedersen et al., 2001). On the other hand, IL-6 is also considered a pro-inflammatory cytokine. Therefore, further investigations are needed to identify the exact role of IL-6 in muscle and other influenced tissues (Abeywardena et al., 2009).

Leukemia inhibitory factor belongs to the IL-6 superfamily (Rose-John et al., 2006) and is secreted by hypertrophic muscle (Spangenburg and Booth, 2006; Serrano et al., 2008; Guerci et al., 2012). It is also released upon resistance training in human muscle and in electrically stimulated cultured human myoblasts (Broholm et al., 2011). Studies in rodents demonstrated that production of IL-6 and leukemia inhibitory factor help to regenerate muscle tissue after injury by activating satellite cells (Barnard et al., 1994; Kurek et al., 1996, 1997; Zhang et al., 2013).

Irisin is a hormone-like myokine secreted during exercise (Boström et al., 2012). It plays an important role in bone-muscle cross talk, and supposedly influences both tissues (Colaianni et al., 2017). This might explain why diseases like osteoporosis and sarcopenia are linked to each other (Reginster et al., 2015). Studies suggest that myokines like IL-6, IL-8, and IL-15 indirectly influence bone via acting on other tissues, while irisin affects bone tissue directly by increasing the differentiation of osteoblasts *in vitro* as well as enhancing cortical bone mass *in vivo* (Colaianni et al., 2014, 2015, 2017; Colaianni and Grano, 2015). Irisin also reduces body weight when administered to obese patients (Colaianni et al., 2017). Another study showed that irisin uptake reduces body weight due to increased adipocyte and glucose metabolism and even elevated oxygen intake levels in an animal model (Boström et al., 2012). Irisin has the potential to transform white adipose tissue into brown adipose tissue, which is metabolically very active. This is supposed to ameliorate obesity and is called browning (Bartelt and Heeren, 2013). Furthermore, Colaianni et al conducted a study in which they analyzed conditioned media from muscle cells of mice performing exercise training. They found that Irisin levels in the media caused a stronger differentiation of bone marrow stromal cells into osteoblasts (Colaianni et al., 2014).

Another myokine, Meteorin-like 1, also induces adipose tissue browning (Rao et al., 2014). The myokines IL-8 and Fstl-1 both induce angiogenesis (Nielsen and Pedersen, 2007), the latter by inducing endothelial cell-mediated neovascularization in ischemic tissue (Ouchi et al., 2008).

IL-15 as well as IL-15 receptor alpha are involved in anabolic/catabolic regulation of skeletal muscle tissue (Quinn et al., 1995; Quinn, 2002; Furmanczyk and Quinn, 2003; Riechman et al., 2004; Busquets et al., 2005; Pistilli et al., 2007). IL-15 enhances the expression of myosin heavy chain in differentiating myocytes (Quinn et al., 1995) and myotubes (Quinn, 2002; Furmanczyk and Quinn, 2003). One session of resistance exercise is sufficient to increase IL-15 levels in trained and untrained humans (Riechman et al., 2004). Furthermore, Quinn et al found high levels of IL-15 to reduce fat mass and therefore lower adiposity in mice (Quinn et al., 2008).

In 2012, Seldin et al. identified another myokine called myonectin. Higher levels of myonectin were secreted into the media by differentiated C2C12 compared to non-differentiated cells. Furthermore, exercise elevated the expression of myonectin in muscle and it is putatively involved in the cross talk between muscle and other tissues like liver and adipose tissue (Seldin et al., 2012).

Another exercise-induced myokine, musculin, is activated by calcium signaling via the AKT pathway (Subbotina et al., 2015). Its function is to enhance mitochondrial biogenesis, which improves physical perseverance (Nishizawa et al., 2004; Subbotina et al., 2015).

SPARC is a myokine found in humans and mice and secreted during muscle contraction (Songsorn et al., 2016). Catoire et al. first identified SPARC to be released upon exercise training through secretome analysis (Catoire et al., 2014). SPARC affects many crucial mechanisms in the cell such as regulation of cell shape, differentiation and adhesion (Murphy-Ullrich and Sage, 2014). SPARC additionally affects insulin secretion in humans (Harries et al., 2013) and erythropoiesis in mice (Luo et al., 2012). Interestingly, the duration of the exercise seems to be more important than the intensity of the exercise for its secretion (Songsorn et al., 2016).

### Disease models

To this day there are no effective cures for muscular dystrophies, hence there is an urgent need for models mimicking them. Novel biomimetic disease modeling platforms could offer a way to understand and study underlying mechanisms of such diseases and furthermore to test potential treatment options. Muscular dystrophy disease models have been used to study the underlying mechanisms, course of the disease over time, as well as therapeutic agents. Some of these will be discussed in the next sections. Animal models are the cornerstone of research on elucidating the mechanisms underlying dystrophies and on developing new treatment strategies. To date, there are around 50 *in vivo* animal models for studying muscular dystrophies in various species ranging from invertebrates (e.g., *Caenorhabditis elegans*), non-mammalian vertebrates, especially zebrafish (Guyon et al., 2007), to mammals (e.g., mice, rats, dogs, and pigs) (McGreevy et al., 2015). The most commonly utilized mammalian DMD models are the mdx mouse model, the mdx utrophin double mutant mouse model (mdx:utrn^−/−^) and the canine x-linked MD model (cxmd) (Banks and Chamberlain, 2008). A frequently used model system is the *mdx* mouse that carries a mutation in the dystrophin gene, resulting in a DMD phenotype. However, there are significant differences in the course of the disease in human DMD patients and *mdx* mice regarding characteristics, such as lifespan, severity, timeline, body weight, impact on other physiological functions and many more (Partridge, 2010). Canine DMD models offer a way to overcome these obstacles, as they present fewer differences to the human DMD pathology regarding the aforementioned characteristics. Furthermore, dystrophic dogs are more suitable for studies using gene therapy approaches than mice, since the former presents a closer simulation of the human immune response toward introduction of vectors for gene repair and replacement (McGreevy et al., 2015). Thus, development of new strategies to introduce vectors evading the immune system is facilitated (Duan, 2015).

### *In vitro* skeletal muscle disease models

Despite the immense amount of knowledge gained on the pathophysiology of skeletal muscle diseases, animal models entail certain disadvantages and ethical considerations. They cannot recapitulate the exact manifestation of the disease in regard of physiological, biochemical and clinical conditions as they appear in the human body. This has prompted research trying to find appropriate time and cost effective alternatives (Banks and Chamberlain, 2008; Benam et al., 2015). Furthermore, results gained from *in vivo* drug testing setups frequently fail when translated to the clinics, due to major differences in underlying molecular mechanisms between different species. *In vitro* human disease models are a potential way to overcome these limitations. They can more closely mimic human pathological conditions concerning tissue and organ specific cell types (Benam et al., 2015) through the possibility of using patient-derived cells, which reflects the patient's individual skeletal muscle physiology and the disease progression in the dystrophic state (Smith et al., 2016). Moreover, it is possible to change single parameters within these systems and study the resulting effects, which constitutes another advantage of *in vitro* disease modeling (Bersini et al., 2016).

In general, miniaturizing disease models has gained attraction in the past years, as it allows reduction in cell number and reagents (and, thus, overall costs), while maintaining the quality of results. Additionally, the assessment of results and provision of external stimuli can be carried out in a precise and controlled manner. Thus, research on microfluidic devices for disease modeling has emerged in recent years (Bersini et al., 2016). One microfluidic system by Ferreira et al used a different approach in modeling dystrophy. Instead of using cells with a diseased phenotype, they established a device that mimicked the cellular environment of dystrophies. This was done by using different ECM compositions and applying a concentration gradient of basic FGF (bFGF), which is known to be released upon muscle injury. Thereby, it was possible to assess the influence of bFGF and different substrates on myoblast recruitment in normal or DMD simulating environments (Ferreira et al., 2015).

Another miniaturized 2D *in* vitro disease model was published by Serena et al, who created myotubes derived from primary myoblasts from healthy donors as well as patients suffering from DMD cultured on polyacrylamide hydrogels. This was achieved through adequate substrate design, including appropriate mechanical properties (i.e., a Young's modulus of 15 kPa). Furthermore, micro-patterning the substrate in parallel lanes to enhance myotube alignment and coatings with the adhesion molecules laminin, fibronectin and Matrigel® were utilized as well. Thereby, it was possible to generate myotubes positive for myosin heavy chain II and α-actinin that developed a highly ordered sarcomeric patterning. Furthermore, myotubes generated from healthy donors exhibited dystrophin expression. This is a key aspect for assessing the functionality of DMD therapies, as they often aim at restoring dystrophin expression (Serena et al., 2010). The basic principle of this test system was recently used to study the potential of mesoangioblasts in DMD treatment as they ameliorated dystrophin distribution in DMD myoblasts (Serena et al., 2016).

The course of dystrophies varies widely from one patient to another, as the mutations causing the disease are very heterogeneous, ranging from severe forms completely lacking dystrophin to a partially functioning truncated form of the protein. This variance cannot be considered in animal or standard *in vitro* models. Creating iPSCs from patient-derived cells offers a solution to this problem, since it allows direct comparison of the pathological phenotype of the patient and the cultured cells. This makes drug screening results and the evaluation of specific genetic aberrations more reliable. Therefore, they present a promising tool for modeling a variety of diseases. Also, recent advances in the field of iPSC research have boosted the efficiency of reprogramming. Myogenic progenitor-derived iPSCs showed good engraftment after transplantation, were able to regenerate myofibers and could repopulate the stem cell niche (Darabi et al., 2012; Meregalli et al., 2014; Kodaka et al., 2017). Nevertheless, this approach also bears certain disadvantages such as the lengthy processes involved in generating iPSCs and inducing differentiation into iPSC-derived myogenic progenitors, or the need to integrate so-called reprogramming factors, which could have unknown implications on the phenotype of the disease (Smith et al., 2016). Tanaka et al. were able to create myotubes from human iPSCs derived from Miyoshi Myopathy patients through inducible MyoD1 expression. These myotubes exhibited hallmarks of the disease, such as the role of Dysferlin during this disease. A lack of Dysferlin expression led to inefficient membrane repair, which could be overcome by an induced overexpression of Dysferlin, rescuing dystrophic myotubes and leading to a healthy phenotype. These results suggest that this model has the potential to shed light on the pathology of the disease, and may be applicable to other types of dystrophies (Tanaka et al., 2013). Another study using human iPSCs from DMD and Becker MD patients was published by Abujarour et al. Human iPSCs were subjected to MyoD1 overexpression, inducing myogenic commitment and finally yielding myotubes. To investigate whether this model has the potential to be used for drug testing, dystrophic myotubes were subjected to IGF-1 and Wnt7a treatment, factors that elicit skeletal muscle hypertrophy. A treatment with these two factors resulted in significant increase in fiber diameter, suggesting usability of this model for drug testing (Abujarour et al., 2014). Nevertheless, to date these models have not been used to test drugs or other therapies for DMD.

However, it is not possible to accurately mimic the complex organization of tissues *in vivo* using 2D disease models. Thus, drug-screening results gained from these systems cannot directly be translated for the use in clinical studies. To overcome the limitations of 2D cell-based systems, more recent research has focused on the development of 3D systems that more adequately reflect the *in vivo* situation (Nam et al., 2015), where cells can interact with the matrix they are embedded in and form 3D structures (Bersini et al., 2016). A 3D drug testing platform was established by Madden and colleagues using human primary myoblasts grown in so-called myobundles generated by incorporation in fibrinogen and Matrigel® frames using polydimethylsiloxane molds. The bundles differentiated into chemically and electrically responsive muscle-like constructs capable of contraction. To prove their suitability for drug screening, the myobundles were treated with three different drugs, namely statins that induce muscle weakness, chloroquine that induces autophagy and clenbuterol which increases hypertrophy in low doses but leads to apoptosis and necrosis at higher concentrations. Overall, treatment with these compounds resulted in the expected outcomes. Therefore, this model appears suitable for drug testing. However, its usability as a disease model to study the pathophysiology of dystrophies remains to be established, as it has only been examined with cells derived from healthy donors (Madden et al., 2015). Thus, there is only one actual *in vitro* skeletal muscle disease model reported in 3D so far. This model used dystrophic myoblasts from *mdx* mice that were incorporated in natural hydrogels (collagen type I or fibrin) that were cast around posts. The resultant myotubes were electrically stimulated and contractile force generation was measured. In addition, 31 compounds that have the potential to serve as DMD drugs were screened by measuring changes in force generation upon treatment. Since this system works semi-automatically and in a 96-well culture format it is considered a potential high-throughput system for testing novel drugs for MD treatment. The major drawback of this model, however, is that it used murine cells. Thus, the results do not account for possible differences in drug response between humans and mice. Furthermore, the phenotype of the engineered constructs appeared to be closer to neonatal than adult according to the myosin heavy chain profiles (Vandenburgh et al., 2008).

In summary, there is still a great need for further research in the field of 3D skeletal muscle disease modeling. The creation of mature and functional *in vitro* muscle constructs could help enhance our fundamental understanding of the skeletal muscle physiology. Hence, the next step would be to create appropriate and translatable disease model systems to bring *in vitro* research one step closer to the *in homine* situation.

## Future perspectives of skeletal muscle tissue engineering

When it comes to SMTE approaches, the fact that 2D culture systems behave fundamentally different from 3D systems has often been overlooked. Hence, results from 2D experiments may not be directly compared or even translated to 3D settings. Identifying applicable treatment options will require engineering of functional 3D muscle tissue constructs. In this respect, several questions need to be addressed: (I) When does one look at gene expression levels or signaling pathways involved in muscle development or differentiation? (II) What are representative time points for the evidence of mature and functional muscle tissue? (III) Which analytical tools and methods can be applied for the morphological and functional assessment of skeletal muscle tissue constructs? (IV) How can a given biomaterial recapitulate the physiological environment supporting the myogenic potential of the cells?

**Figure 3:**
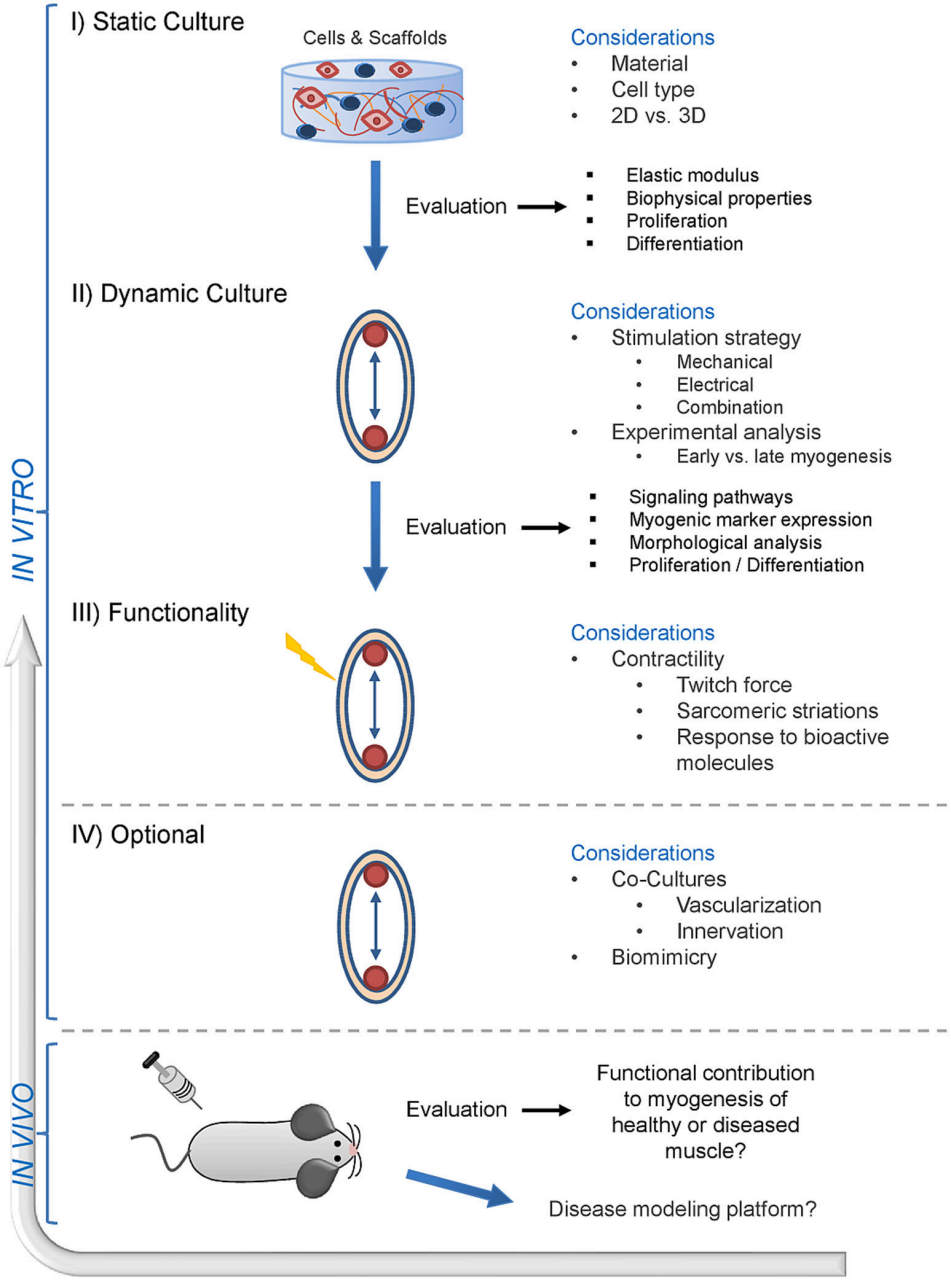
Envisioned future of skeletal muscle tissue engineering—a suggested workflow. This schematic presents a skeletal muscle tissue engineering workflow including stage-specific experimental considerations. Initially, the compatibility of biomaterials with potent myogenic cells has to be evaluated. This first step also involves the decision whether the cells will be cultured and grown in a 2D (monolayer on a pliant matrix) or 3D (encapsulation into a pliant matrix) environment. This still represents a static cell culture, where only the first steps in the SMTE approach are addressed. Evaluation of the biophysical matrix properties, biocompatibility and effects of the biomaterial on cell proliferation/differentiation can be evaluated via this process. The second step involves dynamic culture of the evaluated biomaterial and cells, where the main consideration is which stimulation strategy will be implemented into the culture system—ranging from mechanical to electrical stimulation or a combination of both. The third step addresses the functional analysis of the engineered muscle construct via twitch force measurements. At this point, contractile muscle constructs can furthermore be tested for their response to drugs with known effects, which is a prerequisite for later application of engineered muscle tissue in drug screening studies. An ideal setup would involve co-cultures to engineer muscle tissue with built-in vascular and neuronal structures to further enhance muscle maturity and contractility. After successful *in vitro* evaluation, the final step is the translation into animal models to test for the contribution of the engineered muscle to myogenesis and regeneration in healthy and/or diseased muscle. Ultimately, the knowledge gained from *in vivo* experiments can also be transferred back to *in vitro* setups for the generation of disease models.

Therefore, there is urgent need for standardized dynamic 3D model systems to enable comparability of results. Additionally, careful deliberation of the choice of biomaterial, cell type and the external stimuli, prior to the start of the actual experimental SMTE approach, may help to improve the outcome and save valuable time (Figure 2). The field of SMTE would greatly benefit from a workflow of criteria, factors, and analytical methods, which could be utilized by researchers globally. Here we provide a putative example of such a workflow that displays different experimental and developmental stages in *in vitro* SMTE culture systems, and produces results that are translatable to *in vivo* settings (Figure 3). It suggests analytical tools for endpoint analysis and evaluation of requirements for achieving SMTE constructs with desirable properties (e.g., determination of elastic modulus, activation of involved signaling pathways and expression of myogenic markers and functional characteristics). Optimization of dynamic culture conditions comprises a thorough cell biological analysis including investigation of signaling pathways involved in myogenesis, muscle hypertrophy and proliferation, myogenic gene expression profiling, morphological analysis through immunofluorescence staining for contractile proteins, calculation of the fusion index and the quantification of sarcomeric striations—the latter indicating a certain degree of muscle maturity. Finally, environmental culture conditions should be fine-tuned, e.g., applying external stimuli including appropriate training. Such stimuli are commonly applied via bioreactor systems, which contribute to the desired outcome of engineering 3D skeletal muscle constructs by recapitulating physiological or pathophysiological muscle states. A unified SMTE approach following certain design criteria would render results between groups more comparable, possibly accelerating and streamlining new therapeutic discoveries and advancements in the field of SMTE.

## Conclusion

Numerous sophisticated SMTE strategies exist, ranging from basic 2D to complex dynamic 3D setups, and researchers have a plethora of biomaterials and cell types to choose from. Nevertheless, to date the clear majority of SMTE approaches have failed to achieve broad clinical utility due to several reasons: (I) Systemic elucidation of suitable cell types and biomaterials as well as stimulation protocols (to induce muscle maturation) are still ongoing. (II) The pathomechanisms of a variety of MDs are still poorly understood which limits the clinical success of cell therapeutic approaches. Hence, model systems for developmental/mechanistic and pathophysiological studies (disease models) are urgently needed to perform drug screenings for potential new treatment options. Currently, the focus is on finding reliable physiological models to further understand and study the pathophysiological processes in MDs. (III) Although acellular approaches bypass the general risks associated with (stem) cell therapy, many seemingly promising biomaterials have ultimately failed to meet the physical and native requirements to drive muscle regeneration.

ESC- and iPSC-derived myogenic precursors are increasingly used for drug screening purposes in disease models, while immortalized cell lines are used for initial testing of novel biomaterials and/or bioreactor systems. In an optimal scenario, autologous primary muscle (stem) cells directly derived from the patient would be used for personalized therapeutic approaches or disease models that involve the use of either undifferentiated or preconditioned cells. Although the current pool of applicable cells permits many different methodologies, each cell type has its limitations. However, advances in cell biology will establish adequate culture conditions in the future which will ideally diminish the phenotypic changes of cell types suitable for SMTE during *ex vivo* culture. Biomaterial systems that can serve as artificial satellite cell niches have already improved the efficiency of cell grafting in *in vivo* studies, and a more thorough evaluation of the satellite cell niche composition and microarchitecture will further improve current cell-based therapies. Finally, strategies for *in vitro* pre-vascularization and innervation will likely enhance the functional contribution of engineered muscle transplants to repair muscle *in vivo*. In addition, co-culture systems will allow studies on the interface between the different cell types in the muscle construct. Furthermore, myokines might offer novel therapeutic opportunities in the future, due to their positive effects on muscle as well as on other tissues. In *in vitro* culture systems, they might also be useful as supplements which can act as supportive factors for myogenesis, thereby improving the myogenic outcome of engineered muscle tissue constructs.

Therefore, elevating SMTE to the next level will require a thorough re-evaluation of biomaterial and cell sources as well as fine-tuning of stimulation techniques. Additionally, taking the above-mentioned criteria into account and implementing them into current research strategies will yield novel skeletal muscle (disease) model systems helping to improve therapeutic approaches to finally translate them into clinical setups.

## Author contributions

CF: PI of the project, preparation of the manuscript, design of the figures; BM: preparation of the manuscript and tables; JT: contributed to cell part and disease model part of manuscript, revising the manuscript; PH: preparation of the manuscript and help in figure design, revising the manuscript; OS: PhD supervisor of BM, corrections of the manuscript and input on figures and tables; DR: finances the project, head of department of biochemical engineering, corrections of the manuscript and input on figures and tables.

### Conflict of interest statement

The authors declare that the research was conducted in the absence of any commercial or financial relationships that could be construed as a potential conflict of interest. The reviewer SG and handling Editor declared their shared affiliation.
